# Synergistic In Vitro Interaction of Isavuconazole and Isoquercitrin against *Candida glabrata*

**DOI:** 10.3390/jof8050525

**Published:** 2022-05-20

**Authors:** Petra V. Schwarz, Ilya Nikolskiy, Eric Dannaoui, Frank Sommer, Gert Bange, Patrick Schwarz

**Affiliations:** 1 Center for Invasive Mycoses and Antifungals, Faculty of Medicine, Philipps University Marburg, D-35043 Marburg, Germany; muelle6o@students.uni-marburg.de (P.V.S.); ilian@students.uni-marburg.de (I.N.); 2 Center for Synthetic Microbiology (SYNMIKRO), Department of Chemistry, Philipps University Marburg, D-35043 Marburg, Germany; gert.bange@synmikro.uni-marburg.de; 3 Unité de Parasitologie-Mycologie, Hôpital Européen Georges-Pompidou, F-75015 Paris, France; eric.dannaoui@aphp.fr; 4 Dynamyc Research Group (EA 7380), Faculté de Médecine de Créteil, Université Paris-Est-Créteil-Val-de-Marne, F-94010 Créteil, France; 5 Faculté de Médecine, Université de Paris, F-75006 Paris, France; 6 Department of Microbiology, University Hospital Marburg, D-35032 Marburg, Germany; frank.sommer@med.uni-marburg.de; 7 “Molecular Physiology of Microbes” Group, Max Planck Institute for Terrestrial Microbiology, D-35043 Marburg, Germany; 8 Department of Internal Medicine, Respiratory and Critical Care Medicine, University Hospital Marburg, D-35033 Marburg, Germany

**Keywords:** antifungal combination, *Candida*, isoquercitrin, EUCAST, in vitro, isavuconazole

## Abstract

In vitro interactions of broad-spectrum azole isavuconazole with flavonoid isoquercitrin were evaluated by a broth microdilution checkerboard technique based on the European Committee on Antimicrobial Susceptibility Testing (EUCAST) reference methodology for antifungal susceptibility testing against 60 *Candida* strains belonging to the species *Candida albicans* (*n* = 10), *Candida glabrata* (*n* = 30), *Candida kefyr* (*n* = 6), *Candida krusei* (*n* = 5), *Candida parapsilosis* (*n* = 4), and *Candida tropicalis* (*n* = 5). The results were analyzed with the fractional inhibitory concentration index and by response surface analysis based on the Bliss model. Synergy was found for all *C. glabrata* strains, when the results were interpreted by the fractional inhibitory concentration index, and for 60% of the strains when response surface analysis was used. Interaction for all other species was indifferent for all strains tested, whatever interpretation model used. Importantly, antagonistic interaction was never observed.

## 1. Introduction

Invasive candidiasis is a life-threatening disease associated with a high mortality rate of approximately 40% [1]. Ninety-five percent of invasive *Candida* infections are caused by only five different *Candida* species: *Candida albicans*, *Candida glabrata*, *Candida krusei*, *Candida parapsilosis*, and *Candida tropicalis* [2]. The majority of infections worldwide are still caused by *C. albicans* [3], but non-*albicans Candida* species can represent up to 60% in the United States, or in parts of Europe [4,5]. The second most common species found in the United States and Central Europe is *C. glabrata* [6]. First-line therapy for invasive candidiasis are echinocandins. Azoles for primary therapy are not recommended anymore, but can be used as step-down therapy in case the infecting organism is susceptible [7]. *C. parapsilosis* has an intrinsic, decreased susceptibility to caspofungin [8]; *C. krusei* even has an intrinsic fluconazole resistance [9]. Echinocandin resistance in *C. glabrata* and other *Candida* species can be acquired by mutations in the glucan synthase encoding genes, *FKS1* and *FKS2* [10,11]. Echinocandin resistance is emerging, particularly in *C. glabrata*, with variable rates of 10–13% in the United States [12,13], 2.5% in Canada [14], 2.7% in Denmark [15], and 1.1–10% in Ibero-America [16], but can be as high as 48%, as reported in Germany. Of the 176 *C. glabrata* isolates submitted to the National Reference Center for Invasive Fungal Infections from 2015 to 2019, 84 were anidulafungin resistant, based on the European Committee on Antimicrobial Susceptibility Testing (EUCAST) susceptibility testing methodology. Seventy-one of these strains harbored *FKS* gene mutations. Over one-third of the echinocandin-resistant strains additionally displayed concomitant fluconazole resistance [17]. The high mortality rate, the lack of efficacy in monotherapy for some difficult-to-treat infections [18], and the emergence of antifungal resistance, especially among *C. glabrata*, underscore the need for alternative approaches. A promising strategy is the use of antifungals in combination. The main advantages of antifungal combinations are the possibility to increase efficacy [19], to reduce toxicity, to improve the pharmacokinetics of the molecules [20], and to overcome resistance [21]. However, using the common antifungals, no promising combination for the treatment of invasive candidiasis has been found [22]. As shown for *Candida* [23,24,25], *Aspergillus* [26,27], and Mucorales [28], combinations of non-antifungals with antifungals can also exhibit synergy. Isavuconazole is a broad-spectrum azole drug with potent in vitro activity against *Candida* blood-stream isolates, particularly against *C. albicans* and *C. glabrata* [29]. However, despite proven efficacy for the treatment of aspergillosis [30], isavuconazole could not prove non-inferiority compared with caspofungin as a primary treatment of invasive candidiasis [31]. Moreover, as the mechanisms of resistance for fluconazole and isavuconazole are similar [32], it has to be anticipated that isavuconazole resistance could also be a problem for the treatment of invasive candidiasis, but due to its limited use for treating invasive candidiasis, the rate of resistance is unknown. Isoquercitrin is a flavonoid which has shown fungicidal activity against *C. albicans* [33], which makes it an interesting partner to test in vitro combinations. Therefore, the purpose of this study was to evaluate if isoquercitrin can enhance the in vitro activity of isavuconazole against common *Candida* species, including *C. glabrata*, assessed by a checkerboard technique based on the EUCAST methodology for antifungal susceptibility testing.

## 2. Materials and Methods

### 2.1. Strains

This study included a total of 60 clinical *Candida* strains belonging to six different species. Included were 10 *C. albicans*, 30 *C. glabrata*, 6 *Candida kefyr*, 5 *C. krusei*, 4 *C. parapsilosis*, and 5 *C. tropicalis* strains. Three strains belonged to international collections; two of these strains belonged to the American Type Culture Collection and the other to the Deutsche Sammlung von Mikroorganismen und Zellkulturen (DSM; https://www.dsmz.de (accessed on 1 May 2022)). The other 57 strains were obtained from the Department of Microbiology of the University Hospital Marburg. The strains not belonging to international collections were all identified to the species level by the sequencing of the complete Internal Transcribed Spacer (ITS)1–5.8S-ITS2 region, as described elsewhere [34]. All sequences were deposited at GenBank under the accession numbers OL351325 to OL351353, OL351355, and OL351356 [24], under OM859334 to OM859338 [23], and under ON391951 to ON391970. Each batch of microplates was tested by the quality control strains *C. krusei* ATCC 6258 and *C. parapsilosis* ATCC 22019, as recommended by EUCAST.

### 2.2. Drugs

Isavuconazole powder was obtained from Pfizer (Berlin, Germany). The stock solution of isavuconazole was prepared in dimethyl sulfoxide (DMSO) at a concentration of 3200 µg/mL. Isoquercitrin was purchased as powder from Merck (Darmstadt, Germany), and was solved in DMSO at a concentration of 12,800 µg/mL. Both stock solutions were kept at −25 °C until use.

### 2.3. Medium Preparation

All experiments were carried out in Roswell Park Memorial Institute 1640 (RPMI) medium (with L-glutamine and pH indicator, but without bicarbonate) (Merck). The medium was prepared in double strength and contained 2% (*w/v*) of D-Glucose and the buffer 3-(*N*-morpholino) propanesulfonic acid (Merck) at a final concentration of 0.165 mol/L. After adjustment of the pH to 7.0 with 2 molar NaOH, the medium was sterilized by vacuum filtration through a 0.22 µm pore-sized filter (Merck).

### 2.4. Microplate Preparation

All experiments of this study were carried out in Nunclon^TM^ delta surface 96-well microtiter plates for adherent cells (Thermo Fisher Scientific, Darmstadt, Germany). A two-dimensional checkerboard was used to evaluate the combination of isavuconazole with isoquercitrin [35]. Therefore, an antifungal susceptibility testing protocol, modified for broth microdilution combination studies, based on the EUCAST guidelines was used [36]. Each drug was two-fold serial diluted in the double strength RPMI medium. As the tested *Candida* species exhibited different susceptibility profiles to isavuconazole, two kinds of microplates were prepared. To test *C. albicans*, *C. kefyr*, *C. parapsilosis,* and *C. tropicalis* strains, the final concentrations for isavuconazole on the microplates ranged from 0.00006 to 0.03 µg/mL, while 0.001 to 0.5 µg/mL was used to test *C. glabrata* and *C. krusei* strains. Isoquercitrin ranged from 1 to 64 µg/mL on all prepared microplates. The last column of the microplates contained only the RMPI medium without drugs and was used as the growth control (positive control). Before the addition of the inoculum, each well of the microplates contained 1% (*v/v*) DMSO.

### 2.5. Inoculum Preparation and Inoculation of Microplates

*Candida* strains were cultured from stocks frozen in 40% (*v/v*) glycerol at 35 °C and 95% humidity on Sabouraud dextrose agar slants, supplemented with chloramphenicol and gentamicin (Bio-Rad Laboratories, Feldkirchen, Germany). Twenty-four hours before the experiments, the strains were subcultured again under the same conditions. After subculture, yeast cells were transferred to sterile tubes, containing pure sterile water, by using inoculation loops. The final inoculum was adjusted to 2 × 10^5^ colony-forming units (CFU)/mL after counting the cells in a hemocytometer. Directly after adjustment, Eppendorf Xplorer plus (Eppendorf, Hamburg, Germany) electric multichannel pipettes were used to distribute 100 µL of the inoculum into each well of the microplates. To ensure the inoculum size, the final inoculum was further diluted to 1:10 and 50 µL were spread once on the Sabouraud dextrose agar plates with a sterile Drigalski spatula. The CFU were counted 24 hours after incubation. The microplates were incubated for 24 h at 35 °C and 95% humidity and the optical densities were read spectrophotometrically at a wavelength of 530 nm, using a MultiSkan FC spectrometer (Thermo Fisher Scientific). Before spectrophotometric reading, all microplates were shaken for 2 min at 1100 rpm with the PMS-1000 Microplate Shaker (Grant Instruments, Shepreth, UK) to dissolve the yeast colonies in the wells. A blank plate, in which each well was inoculated with 100 µL of sterile distilled water, was incubated under the same conditions as mentioned above (negative control). The blank plate was read spectrophotometrically at 530 nm and the optical density values were subtracted from the values of the microplates inoculated with the *Candida* strains. The resulting optical density values were transformed into the percentage of growth compared with the growth control, and used for the calculation of the results. Combination experiments were run twice.

### 2.6. Interpretation of the Results by Fractional Inhibition Concentration Index

Fifty percent of inhibition was chosen as an endpoint for the determination of the MICs alone of both drugs and in combination. High off-scale MICs were converted to the next log_2_ dilution. The fractional inhibition concentration index (FICI) was calculated the following way: FICI = (MIC_isavuconazole in combination_/MIC_isavuconazole alone_) + (MIC_isoquercitrin in combination_ /MIC_isoquercitrin alone_). For FICI values ≤0.5, synergy was concluded; indifference was concluded if the values were >0.5 to 4, and antagonism was concluded if the values were >4 [37].

### 2.7. Interpretation of the Results by Response Surface Analysis

Response surface analysis is an MIC and inhibition endpoint independent method to analyze checkerboard data. It allows the determination and visualization of the drug interactions on the complete surface of the microplate. FICI analyzes are limited to the evaluation of the interaction only for the MICs in combination. To analyze the checkerboard data, special programs are used; in the case of this study, all calculations were done by the Combenefit software [38]. First, the program calculates dose–response curves for the drugs alone, which are based on the growth rates in the wells of the single molecules. From the dose–response curves of the two drugs alone, according to the chosen theoretical model (this study uses the Bliss independence model), a dose–response surface with an indifferent interaction is calculated. The Bliss independence model is based on the hypothesis that drugs act independently from each other. Second, to evaluate the interaction of the combination, the program compares the experimentally obtained dose–response surface with the calculated indifferent dose–response surface. Synergy is observed if the experimentally obtained dose–response surface lies below the calculated dose–response surface. This corresponds to less growth on the microplate, compared with an indifferent interaction. If more growth is obtained on the plate compared with an indifferent interaction, which means that the experimentally obtained dose–response surface lies above the calculated indifferent dose–response surface, antagonism is concluded. Third, the program calculates the SUM-SYN-ANT metric to quantitatively assess the interaction of the drugs. The SUM-SYN-ANT metric is the sum of all the dose–response surface values lying below the calculated indifferent dose–response surface (SYN-SUM), minus the sum of all values lying above (ANT-SUM). Broth microdilution techniques have an intrinsic variability which makes the definition of a threshold necessary. This threshold defines the values for which the interaction of the combination is assumed to be indifferent. Threshold determination can be done experimentally by combining the active molecules with themselves. In the case of this study, the active molecule isavuconazole was tested with itself on the two-dimensional checkerboard with two-fold serial dilutions using the preparation, incubation, and analyzation protocol described above. The quality control strain *C. krusei* ATCC 6258 was used for threshold determination. The strain was tested in triplicate with the highest isavuconazole of 0.12 µg/mL in both axes. Based on these results, synergy was assumed when the SUM-SYN-ANT was ≥56.0%, and antagonism when ≤−56.0%. Between −56.0 and 56.0%, indifference was concluded. For the determination of the SUM-SYN-ANT metric of the tested strains, the data of both runs were combined.

## 3. Results

**Figure 1:**
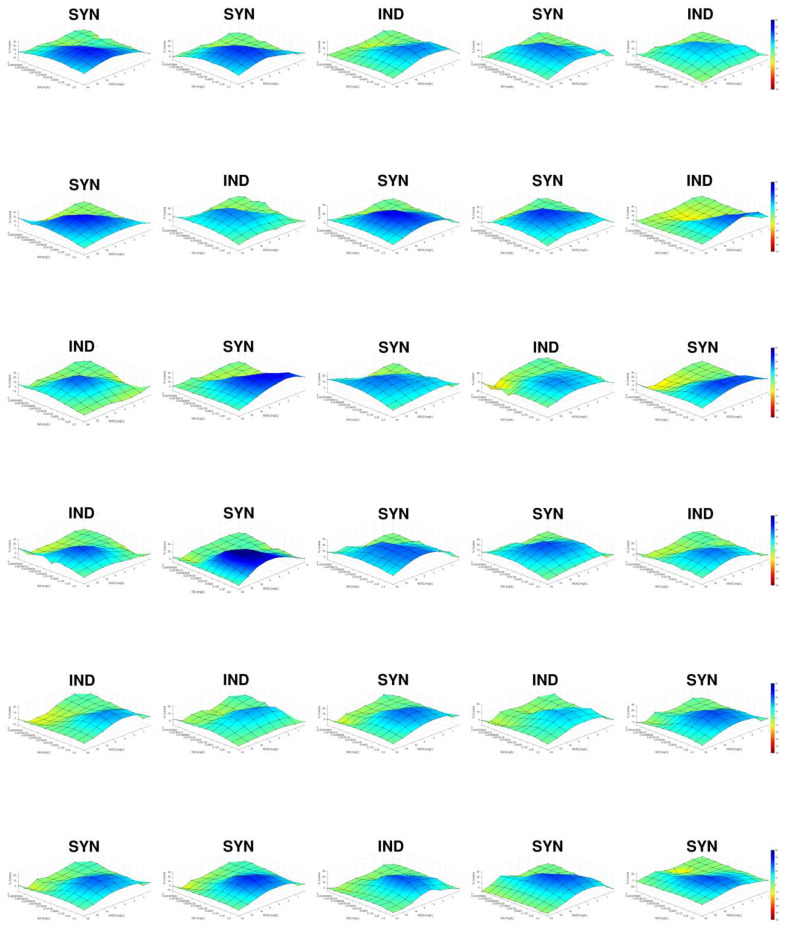
Synergy distribution for the combination of isavuconazole with isoquercitrin against all *C. glabrata* strains tested. Always from the left to the right, first row: V2105272, V2105282, N2101711, V2105636, DSM 70614; second row: U2105834, V2105576, N2102530, U2106503, U2106602; third row: U2106664, U2106745, U2107113, U2107210, U2107214; fourth row: V2107409, N2102703, N2102712, N2102714, U2107517; fifth row: U2107630, U2107836, V2108007, V2108459, B2109750; last row: A2100553, U2107634, U2107796, U2108032, U2107634. The mode of interaction was defined based on the SUM-SYN-ANT metric. IND, indifference; SYN, synergy.

In the first part of this study the combination of isavuconazole with isoquercitrin was screened against a panel of 35 *Candida* strains belonging to common *Candida* species using the EUCAST broth microdilution technique modified for combination studies. The data of these experiments were analyzed by FICI and response surface analysis, and the results are presented in Table 1. Based on these results, 25 additional *C. glabrata* strains were tested and analyzed the same way. The results of these analyses are presented in Table 2. A summary of all results is presented in Table 3. Figure 1 shows the synergy distributions for the combination of isavuconazole with isoquercitrin against all *C. glabrata* strains tested.

The MICs for isavuconazole were within the range of the EUCAST quality control range for this antifungal and are presented in Table 1 (first batch of microplates). The MICs for the quality controls of the second batch of microplates were exactly the same (data not shown). For isoquercitrin, no quality control ranges exist, but the MICs for isoquercitrin of the two quality controls were the same in both batches of microplates.

The 60 *Candida* strains exhibited MICs for isavuconazole ranging from 0.0005 to 1 μg/mL (Table 1 and Table 2) with an MIC_50_, MIC_90_, and geometric mean MIC of 0.06, 0.5, and 0.037 μg/mL, respectively. Isavuconazole MICs ranged from 0.001 to 0.004, 0.008 to 0.016, and 0.0005 to 0.004 µg/mL for *C. albicans*, *C. parapsilosis*, and *C. kefyr*, respectively, or were 0.06 and 0.008 µg/mL for *C. krusei* and *C. tropicalis*, respectively. For the strains of *C. glabrata*, MICs for isavuconazole ranged from 0.125 to 1 μg/mL with an MIC_50_, MIC_90_, and geometric mean MIC of 0.25, 0.5, and 0.28 μg/mL, respectively. When tested alone, isoquercitrin exhibited MICs ranging from 16 of 128 µg/mL for the different species (128 μg/mL being the high off-scale MIC) with an MIC_50_, MIC_90_, and a geometric mean MIC of 64, 128, and 65.5 μg/mL, respectively. Best activity of isoquercitrin was seen against *C. glabrata* with MICs ranging from 16 to 64 µg/mL and a geometric mean MIC of 42.22 µg/mL. Isoquercitrin showed no activity against *C. krusei*, *C. parapsilosis*, and *C. tropicalis*; the MICs of all strains were >64 µg/mL. Almost no activity was seen against *C. kefyr,* with a geometric mean MIC of 101.6 µg/mL. Apart from *C. glabrata*, isoquercitrin exhibited only against *C. albicans* a certain degree of activity with a geometric mean MIC of 73.52 µg/mL. Between the experiments, isavuconazole and isoquercitrin MICs were within +/− 1 log_2_ dilutions in 98.33% of the cases for all *Candida* strains tested (data not shown). Interaction was synergistic for 100% of the tested *C. glabrata* strains (*n* = 30), with FICIs ranging from 0.125 to 0.5 with a geometric mean FICI of 0.25. Interaction against all other tested species was indifferent.

Although synergy was less frequently obtained than by FICI analysis, the interaction of the combination evaluated by response surface analysis was still synergistic for the majority (60%) of the *C. glabrata* stains tested. The SUM-SYN-ANT metric for the synergistic strains ranged from 56.24 to 95.8, with a mean of 76.06. The mean of the SUM-SYN-ANT metric of all strains of *C. glabrata* tested was 64.15. As obtained by FICI analysis, the interaction of the combination evaluated by response surface analysis against all other strains of the tested species was indifferent.

## 4. Discussion

Polyphenols are natural organic compounds comprising of multiple phenol units, found in fruits, vegetables, cereals, and beverages such as red wine or tea [39]. They are secondary metabolites of plants, and involved in the host defense against ultraviolet radiation or pathogens [40]. Epidemiological studies suggested that long-term use of plant polyphenol-rich diets could protect against the development of cancers, cardiovascular diseases, diabetes, aging, asthma, neurodegenerative diseases, and infections [41]. Flavonoids are a class of polyphenols which have a 15-carbon skeleton comprising of two phenyl rings connected over a heterocyclic ring containing embedded oxygen, have the ability to inhibit spore germination in plant pathogenic fungi, and have, therefore, been proposed for use against fungal pathogens of man [42]. Flavonoids have shown antifungal activity against dermatophytes [43,44], human opportunistic filamentous fungi [43,45,46,47,48], and yeasts, including *Candida* species [43,49,50,51,52]. Isoquercitrin is a flavonoid which can be isolated from areal parts of *Aster yomena*, a perennial herb which grows in the southern part of Korea, and is used as a traditional medication to treat inflammation, colds, and bronchial asthma [53]. Apart from its anti-allergic [54], antibiotic [55], anti-hyperlipidemic [56], anti-inflammatory [57], and antioxidant properties [58], it causes fungicidal membrane disturbance [33] and reactive oxygen species (ROS)-mediated apoptosis in *C. albicans* [59], which make the drug an interesting partner to test combinations with antifungals.

The MICs of isavuconazole for the *Candida* species tested in this study were in the same range as described previously [60,61,62]. MICs of isoquercitrin of some of our *C. albicans* strains were in the same range as reported by others who used EUCAST methodology for MIC determination [63]; however, the majority of our strains had higher MICs. Another study which evaluated the activity of different polyphenols isolated from *Pterogyne nitens* found, in accordance with this study, high MICs of isoquercitrin against *C. albicans*, *C. krusei*, and *C. parapsilosis*, but CLSI (Clinical and Laboratory Standards Institute) methodology was used for susceptibility testing.

MICs of isoquercitrin in combination for *C. glabrata* ranged from 2 to 8 μg/mL with a geometric mean MIC of 4 μg/mL. To our knowledge, peak serum levels of isoquercitrin in patients have not been studied so far. In rats, peak plasma levels of about 15 µg/mL were reached after a single intravenous administration of 5 mg/kg of body weight. With doses of 20 mg/kg of body weight, peak plasma levels of even 60 µg/mL have been reached [64]. These levels would have largely been enough against the *C. glabrata* strains tested in this study. However, even if achievable serum levels in patients would be lower, the synergy of the combination could not directly be out of the question, as it has been shown in patients that even lower serum levels than those of the MICs can lead to in vivo synergy [65].

Only one study evaluated the interaction of isoquercitrin with azoles against *Candida*. The combination of isoquercitrin with fluconazole was evaluated by checkerboard against one strain of *C. albicans* and found to be synergistic. The authors further showed that the addition of fluconazole to isoquercitrin increases the effect of isoquercitrin in lowering the activity of the superoxide dismutase and increasing metacaspase activation and DNA condensation, leading to ROS accumulation, oxidative stress, and induction of apoptosis. In the same study, the combination of isoquercitrin with amphotericin or flucytosine was tested against one strain of *C. albicans*, yielding synergy and indifference, respectively [66]. In our study, the combination of isoquercitrin and isavuconazole against 10 *C. albicans* strains evaluated by checkerboard and interpreted by FICI and by response surface analysis exhibited indifference. The different interaction could be specific to the strain used or associated with the different azole.

Against other *Candida* species, the combination of isoquercitrin has never been tested before. We found indifference of the combination of isoquercitrin with isavuconazole for all *C. krusei*, *C. kefyr*. *C. parapsilosis*, and *C. tropicalis* strains tested. In contrast to these results, combination was synergistic against all 30 *C. glabrata* strains tested, when the results of the checkerboard were interpreted by FICI. When the interpretation of the results was carried out by response surface analysis, synergy was still obtained for 60% of the *C. glabrata* strains tested. The discrepancy between the FICI and the response surface analysis results can be explained by the stringent threshold (56.0) used in this study compared with previous works, where lower thresholds have been used [24,25]. Nevertheless, despite the indifference of 40% of the *C. glabrata* results by response surface analysis, the mean of the SUM-SYN-ANT metric of all strains was 64.15, and therefore higher than the threshold.

One of the limitations of this study is that molecular determination of azole resistance or *FKS* gene mutation has not been performed for the *C. glabrata* strains used in this study. This limitation is aggravated by the lack of breakpoint and epidemiological cut-off value definition by EUCAST for isavuconazole against *C. glabrata*. It has been shown that *C. glabrata* strains with proven molecular azole resistance exhibit MICs to isavuconazole of 1 to ≥8 µg/mL [67,68]. In this study, one strain exhibited an MIC of 1 µg/mL. However, it cannot be concluded that this strain owns an azole-resistance mutation because the MICs in the mentioned studies were determined by CLSI methodology and, also, because wild-type strains can exhibit MICs of 1 and even 2 µg/mL to isavuconazole [67]. *C. glabrata* strains resistant to echinocandins are often cross-resistant to azoles. Even if the strain in this study with the MIC of 1 µg/mL would be isavuconazole resistant, it cannot be concluded that the strains are mandatory also echinocandin resistant [69]. Therefore, based on the results of this study, it cannot be concluded that the combination of isavuconazole and isoquercitrin can overcome proven azole or echinocandin resistance.

To summarize, we demonstrated that the combination of isavuconazole with isoquercitrin interacts synergistically against *C. glabrata*. Interaction against *C. albicans*, *C. krusei*, *C. kefyr*, *C. parapsilosis,* and *C. tropicalis* exhibited only indifference, but importantly antagonistic interaction was never observed. These results warrant further animal experiments.

## Figures and Tables

**Table 1:** Interaction of isavuconazole with isoquercitrin against common *Candida* species evaluated by checkerboard and interpretation by fractional inhibitory concentration index and response surface analysis.

Species	Collection Number	Checkerboard MICs (µg/mL)					Response Surface Analysis	
		ISA	ISOQ	ISA/ISOQ	FICI	INTPN	ΣSYN-ANT (ΣSYN; ΣANT)	INTPN
*C. albicans*	V2105126	0.002	128	0.001/64	1	IND	17.47 (19.03; −1.56)	IND
*C. albicans*	N2101578	0.004	128	0.004/1	1.0078	IND	−7.17 (8.40; −15.57)	IND
*C. albicans*	V2105568	0.001	64	0.00006/32	0.5625	IND	27.02 (27.98; −0.96)	IND
*C. albicans*	N2101577	0.002	128	0.002/1	1.0078	IND	34.95 (35.70; −0.75)	IND
*C. albicans*	V2105825iso3	0.001	32	0.0005/16	1	IND	22.97 (23.54; −0.57)	IND
*C. albicans*	ATCC 14053	0.002	32	0.001/1	0.5313	IND	16.83 (18.45; −1.62)	IND
*C. albicans*	V2105529	0.001	32	0.0005/8	0.75	IND	28.19 (28.66; −0.47)	IND
*C. albicans*	V2106139	0.002	64	0.00006/32	0.5313	IND	0.85 (10.26: −9.41)	IND
*C. albicans*	V2106041	0.001	128	0.001/1	1.0078	IND	11.79 (12.27; −0.48)	IND
*C. albicans*	V2106305	0.004	128	0.002/1	0.5078	IND	12.60 (13.34; −0.74)	IND
*C. glabrata*	V2105272	0.5	64	0.06/4	0.1875	SYN	93.42 (93.60; −0.18)	SYN
*C. glabrata*	V2105282	0.5	64	0.016/8	0.1563	SYN	84.19 (84.83; −0.64)	SYN
*C. glabrata*	N2101711	0.125	32	0.03/2	0.3125	SYN	55.66 (56.95; −1.29)	IND
*C. glabrata*	V2105636	0.125	32	0.016/4	0.25	SYN	70.11 (70.56; −0.45)	SYN
*C. glabrata*	DSM 70614	0.125	16	0.03/2	0.375	SYN	46.95 (48.26; −1.31)	IND
*C. krusei*	V2105825iso4	0.06	128	0.06/1	1.0078	IND	22.13 (24.96; −2.83)	IND
*C. krusei*	V2105866	0.06	128	0.06/1	1.0078	IND	−38.29 (1.94; −40.23)	IND
*C. krusei*	V2106177	0.06	128	0.03/1	0.5078	IND	9.70 (19.83; −10.13)	IND
*C. krusei*	V2105920	0.06	128	0.03/1	0.5078	IND	−4.50 (10.81; −15.31)	IND
*C. krusei*	ATCC 6258	0.06	128	0.03/2	0.5156	IND	−30.31 (5.67; −35.98)	IND
*C. parapsilosis*	V2105056	0.008	128	0.008/1	1.0078	IND	−1.10 (11.54; −12.64)	IND
*C. parapsilosis*	V2105223	0.008	128	0.004/16	0.625	IND	−40.04 (9.22; −49.26)	IND
*C. parapsilosis*	B2107379	0.008	128	0.008/1	1.0078	IND	6.03 (13.39; −7.36)	IND
*C. parapsilosis*	ATCC 22019	0.016	128	0.016/1	1.0078	IND	−8.18 (5.27; −13.45)	IND
*C. tropicalis*	V2105128	0.008	128	0.008/1	1.0078	IND	−5.10 (4.14, −9.24)	IND
*C. tropicalis*	V2105245	0.008	128	0.008/1	1.0078	IND	−9.39 (2.91; −12.30)	IND
*C. tropicalis*	V2105598	0.008	128	0.008/1	1.0078	IND	15.03 (16.16; −1.13)	IND
*C. tropicalis*	B1907975	0.008	128	0.008/1	1.0078	IND	−2.05 (6.51; −8.56)	IND
*C. tropicalis*	V2106298	0.008	128	0.008/1	1.0078	IND	−41.98 (1.63; −43.61)	IND
*C. kefyr*	V2106126	0.002	128	0.001/32	0.75	IND	7.71 (12.12; −4.41)	IND
*C. kefyr*	N2101899	0.0005	32	0.0002/8	0.75	IND	9.04 (17.00; −7.96)	IND
*C. kefyr*	N2102541	0.002	128	0.001/8	0.5625	IND	8.52 (18.35; −9.83)	IND
*C. kefyr*	V2107293	0.004	128	0.004/16	1.125	IND	−4.30 (7.64; −11.94)	IND
*C. kefyr*	V2107534	0.0005	128	0.0005/2	1.0156	IND	10.99 (16.08; −5.09)	IND
*C. kefyr*	V2108462	0.002	128	0.002/2	1.0156	IND	10.42 (24.18; −13.76)	IND

FICI, fractional inhibitory concentration index; INTPN, interpretation; SYN, synergy; IND, no interaction; ISA, isavuconazole; ISOQ, isoquercitrin; ATCC, American Type Culture Collection; DSM, Deutsche Sammlung von Mikroorganismen und Zellkulturen.

**Table 2:** Interaction of isavuconazole with isoquercitrin against *C. glabrata* evaluated by checkerboard and interpretation by fractional inhibitory concentration index and response surface analysis.

Species	Collection Number	Checkerboard MICs (µg/mL)					Response Surface Analysis	
		ISA	ISOQ	ISA/ISOQ	FICI	INTPN	ΣSYN-ANT (ΣSYN; ΣANT)	INTPN
*C. glabrata*	U2105834	0.25	32	0.03/4	0.25	SYN	81.95 (83.97; −2.02)	SYN
*C. glabrata*	V2105576	0.25	32	0.03/4	0.25	SYN	53.99 (56.11; −2.12)	IND
*C. glabrata*	N2102530	0.5	32	0.06/2	0.1875	SYN	95.80 (96.38; −0.58)	SYN
*C. glabrata*	U2106503	0.5	32	0.06/2	0.1875	SYN	82.48 (82.62; −0.14)	SYN
*C. glabrata*	U2106602	0.5	16	0.125/2	0.375	SYN	25.99 (37.29; −11.30)	IND
*C. glabrata*	U2106664	0.125	32	0.016/4	0.25	SYN	47.50 (48.14; −0.64)	IND
*C. glabrata*	U2106745	0.25	32	0.03/4	0.25	SYN	78.88 (81.92; −3.04)	SYN
*C. glabrata*	U2107113	0.25	64	0.03/2	0.1563	SYN	76.94 (77.70; −0.76)	SYN
*C. glabrata*	U2107210	0.125	64	0.03/4	0.3125	SYN	51.26 (54.21; −2.95)	IND
*C. glabrata*	U2107214	0.25	64	0.06/8	0.375	SYN	56.24 (61.55; −5.31)	SYN
*C. glabrata*	V2107409	0.25	64	0.03/8	0.25	SYN	55.26 (56.33; −1.07)	IND
*C. glabrata*	N2102703	0.5	64	0.06/4	0.1875	SYN	93.46 (94.17; −0.71)	SYN
*C. glabrata*	N2102712	1	64	0.06/4	0.125	SYN	87.70 (87.85; −0.15)	SYN
*C. glabrata*	N2102714	0.25	64	0.03/4	0.1875	SYN	77.59 (77.66; −0.07)	SYN
*C. glabrata*	U2107517	0.5	64	0.06/8	0.25	SYN	51.55 (53.74; −2.19)	IND
*C. glabrata*	U2107630	0.25	32	0.06/4	0.375	SYN	35.48 (39.20; −3.72)	IND
*C. glabrata*	U2107836	0.25	64	0.03/8	0.25	SYN	39.54 (40.73; −1.19)	IND
*C. glabrata*	V2108007	0.25	64	0.03/8	0.25	SYN	57.34 (58.43; −1.09)	SYN
*C. glabrata*	V2108459	0.25	64	0.06/2	0.2813	SYN	37.39 (39.26; −1.87)	IND
*C. glabrata*	B2109750	0.25	64	0.03/8	0.25	SYN	70.15 (70.26; −0.11)	SYN
*C. glabrata*	A2100553	0.25	64	0.03/8	0.25	SYN	60.71 (61.60; −0.89)	SYN
*C. glabrata*	U2107634	0.5	64	0.06/4	0.1875	SYN	72.52 (73.38; −0.86)	SYN
*C. glabrata*	U2107796	0.25	16	0.06/4	0.5	SYN	55.06 (55.47; −0.41)	IND
*C. glabrata*	U2108032	0.125	16	0.016/4	0.375	SYN	65.49 (66.03; −0.54)	SYN
*C. glabrata*	U2107634	0.5	32	0.06/2	0.1875	SYN	64.03 (65.77; −1.74)	SYN

FICI, fractional inhibitory concentration index; INTPN, interpretation; SYN, synergy; IND, no interaction; ISA, isavuconazole; ISOQ, isoquercitrin.

**Table 3:** Summary of the in vitro interactions of isavuconazole with isoquercitrin against common *Candida* species evaluated by checkerboard and interpretation by fractional concentration index and response surface analysis.

Species (Strains), Interpretation Model	% of Strains with the Following Interaction		
	Synergy	Indifference	Antagonism
*C. albicans* (10), FICI	0	100	0
*C. albicans* (10), RSA	0	100	0
*C. glabrata* (30), FICI	100	00	0
*C. glabrata* (30), RSA	60	40	0
*C. krusei* (5) FICI	0	100	0
*C. krusei* (5), RSA	0	100	0
*C. parapsilosis* (4), FICI	0	100	0
*C. parapsilosis* (4), RSA	0	100	0
*C. tropicalis* (5), FICI	0	100	0
*C. tropicalis* (5), RSA	0	100	0
*C. kefyr* (6), FICI	0	100	0
*C. kefyr* (6), RSA	0	100	0

FICI, fractional inhibitory concentration index; RSA, response surface analysis.

## Data Availability

The datasets of the sequenced *Candida* strains used in this study can be found at GenBank (https://www.ncbi.nlm.nih.gov/genbank/ (accessed on 1 May 2022)) under the accession numbers OL351325 to OL351353, OL351355, and OL351356, under OM859334 to OM859338, and under ON391951 to ON391970.
